# Reproductive and clinical outcomes after minimally invasive isthmocele repair: a comparison of robotic-assisted and laparoscopic approaches

**DOI:** 10.1007/s00404-026-08546-5

**Published:** 2026-07-27

**Authors:** Carolin Schröder, Laura Tascón Padrón, Anna K. Degenhardt, Dominique Koensgen, Lucia A. Otten, Alexander Mustea, Eva K. Egger

**Affiliations:** 1 Department of Gynaecology and Gynaecological Oncology, University Hospital Bonn, Venusberg-Campus 1, 53127 Bonn, Germany; 2 Department of Gynaecological Endocrinology and Reproductive Medicine, University Hospital Bonn, Venusberg-Campus 1, 53127 Bonn, Germany

**Keywords:** Caesarean scar defect, Isthmocele, Niche, Robot-assisted isthmocele repair, Live-birth rate

## Abstract

**Purpose:**

Caesarean scar defect (CSD), also known as an isthmocele or niche, is a common consequence of caesarean delivery, associated with chronic inflammation, abnormal uterine bleeding, pelvic pain, and infertility, though some patients remain asymptomatic. Minimally invasive isthmocele repair (MIIR) has emerged as a promising therapeutic option for symptomatic women and those seeking fertility, yet evidence guiding optimal surgical management remains limited. In particular, data on reproductive outcomes are scarce and largely based on small, heterogeneous case series. To our knowledge, there are no data from direct comparisons between robotic-assisted (RAIR) and conventional laparoscopic (LIR) isthmocele repairs.

**Methods:**

This monocentric retrospective study included patients undergoing RAIR and LIR at the University Hospital Bonn between December 2019 and March 2026. The primary objective was to determine the live-birth rate after MIIR in women attempting conception. Secondary objectives included evaluating patient-reported outcomes, such as symptom relief, and objective outcomes, including changes in niche length, niche depth, and residual myometrial thickness, as well as obstetric outcomes in subsequent pregnancies, and comparisons between RAIR and LIR.

**Results:**

Twenty-eight patients underwent CSD surgery, including 21 RAIR and 7 LIR procedures. Follow-up was available in 82% of patients (median 4 months). No intraoperative or postoperative complications occurred. Among women attempting conception (*n* = 8), the live-birth rate was 75% (6/8), with equal distribution between groups. In the RAIR group, a significant decrease in niche length and niche depth as well as a significant increase in myometrial thickness were observed between pre- and postoperative. In the LIR group, similar trends were noted that did not reach statistical significance. Between-group comparisons showed no significant differences in postoperative imaging outcomes. Symptom improvement was reported in the majority of patients, particularly regarding abnormal uterine bleeding and pelvic pain. Obstetric outcomes included one uterine scar dehiscence and one placenta accreta spectrum disorder (each 3.6%), both occurring after RAIR.

**Conclusion:**

MIIR and especially RAIR represent a promising fertility-preserving and symptom-improving option for patients with CSD.

**Registration:**

This study was registered in the German Clinical Trials Register (DRKS) under the number DRKS00038857 on April 15th 2026.

## What does this study add to the clinical work?

**Table:** 

Adequate tissue apposition and the promotion of optimal wound healing are crucial for successful isthmocele repair, regardless of whether it is accomplished by RAIR or LIR.	

## Introduction

Caesarean scar defect (CSD), also known as uterine isthmoceles or niche, is a pouch-like myometrial defect at the site of a previous caesarean section. Generally, it is characterised by symptoms such as spotting, prolonged menses, dysmenorrhea, dyspareunia, with a negative impact on reproductive outcomes, causing a caesarean scar disorder easily visible on transvaginal ultrasound [1]. The increasing recognition of CSD is likely due not only to rising caesarean delivery rates but also to improved diagnostic imaging and greater clinical awareness. Reported prevalence ranges from 19 to 84%, with higher rates after repeated caesarean sections [2, 3 ] CSD shares pathophysiological features with endometriosis, as ectopic endometrial tissue within the myometrium may induce cyclic inflammation, fibrosis, and neo-innervation [4, 5 ]. Inadequate tissue approximation or insufficient myometrial thickness at the time of caesarean closure may predispose to the development of CSD, whereas optimised multilayer closure techniques may reduce the risk of niche formation [6, 7 ]. In addition, impaired wound healing processes, as described in the context of the injury and repair theory, may contribute to insufficient integration of endometrial tissue into the myometrium, resulting in the formation of a niche-like defect rather than complete scar healing [8]. Niche formation promotes retention of menstrual blood, resulting in abnormal bleeding, and may contribute to retrograde menstruation and peritoneal endometriosis [9]. These mechanisms are associated with chronic pain and increased risk of secondary infertility [10]. However, patients can also be asymptomatic.

Surgical correction has emerged as a relevant therapeutic option for symptomatic patients and for women desiring future fertility. Several minimally invasive techniques have been described, including hysteroscopic, vaginal, laparoscopic, and combined approaches; however, no clear consensus exists on the optimal surgical strategy [11]. In principle, the choice of surgical approach for the correction of an isthmocele defect should be considered of secondary importance, provided that optimal approximation and compression of the wound edges can be reliably achieved. Crucial factors include the placement of full-thickness sutures and the secure tying of knots to ensure adequate tissue apposition and promote optimal wound healing. In this context, insufficient closure techniques or excessive energy use during haemostasis may impair healing and increase the risk of dehiscence or niche persistence. Robot-assisted laparoscopy may facilitate these principles through improved instrument articulation and precision.

Observational studies suggest improvements in myometrial thickness, symptoms, and reproductive outcomes following MIIR, although current evidence remains limited [12–15]. To our knowledge, there are no data from direct comparisons between robotic-assisted (RAIR) and conventional laparoscopic (LIR) isthmocele repairs. The primary outcome of the study is the live-birth rate after MIIR. Secondary outcomes include both subjective (symptom relief) and objective measures (obstetric complications, changes in niche length, niche depth and residual myometrial thickness), as well as a comparison of outcomes between RAIR and LIR.

## Methods and material

In this monocentric retrospective analysis, patients who underwent CSD surgery between December 2019 and March 2026 at the Department of Gynaecology at the University Hospital Bonn were included.

### Patient selection and surgical indication

Before implementation of the robotic surgical programme, CSD repairs were performed by conventional laparoscopy. Following implementation of RAIR at our department in 2022, elective procedures were mainly performed by robotic-assisted laparoscopy, whereas conventional laparoscopy was used mainly for unscheduled or nonelective procedures when the robotic platform was unavailable.

All patients with a sonographically confirmed CSD received individual counselling regarding expectant management and the available surgical options, including hysteroscopic, conventional laparoscopic, and robotic-assisted repair. Counselling included the potential benefits and risks of each approach as well as the limited evidence regarding surgical treatment, particularly in asymptomatic patients. The presence of symptoms was not an obligatory prerequisite for surgery. In asymptomatic patients, the decision to proceed with surgery was made by shared decision-making after comprehensive counselling and consideration of the patient’s reproductive plans and preferences. No predefined threshold of residual myometrial thickness was used as an inclusion criterion or to determine the surgical approach. Hysteroscopic treatment was discussed and offered as an alternative operative option when indicated (for example residual myometrial thickness exceeding 3 mm). Following MIIR, patients were advised to avoid conception for 3–6 months. In subsequent pregnancies, elective repeat caesarean delivery was recommended.

### Ultrasound assessment

The CSD was assessed by transvaginal ultrasound and identified as an indentation of the anterior lower uterine segment at the site of the previous caesarean scar. A niche was defined as an indentation with a depth of at least 2 mm, in accordance with the definition proposed by Jordans et al. [16]. Sonographic measurements obtained in the sagittal plane included maximum niche length, niche depth, and residual myometrial thickness at the thinnest part of the defect. Niche width in the transverse plane and adjacent myometrial thickness were not routinely recorded.

### Follow-up assessment

Clinical data were collected from the electronic patient file, including preoperative and last follow-up (FU) symptom-oriented gynaecological history, examination, transvaginal sonography, and surgical data. Postoperative FU was not performed according to a predefined study-specific schedule but occurred as part of routine clinical care. For the present analysis, patients were additionally recontacted by telephone and invited to attend an updated outpatient FU examination. When an updated assessment could not be obtained because a patient could not be reached or did not attend the proposed appointment, the most recent available postoperative clinical information was used. FU duration was defined as the interval between surgery and the most recent available postoperative clinical information. Information regarding postoperative pregnancies, pregnancy course, and mode of delivery was obtained from both institutional and external medical records.

### Outcome parameters

The primary outcome of the study is the live-birth rate, defined as the proportion of patients who achieve at least one live birth after MIIR at > 24 weeks’ gestation among those attempting conception. This outcome aims to assess the procedure's effectiveness in restoring reproductive potential. Secondary outcomes include both subjective and objective measures. Subjective outcomes comprise self-reported improvement in symptoms such as postmenstrual spotting and pelvic pain. Objective outcomes include changes in residual myometrial thickness and CSD dimensions on imaging (niche length and depth), as well as the incidence of obstetric complications in subsequent pregnancies. Additionally, the outcomes were compared between RAIR and LIR.

### Standard surgical procedure (Fig. 1)

**Fig. 1:**
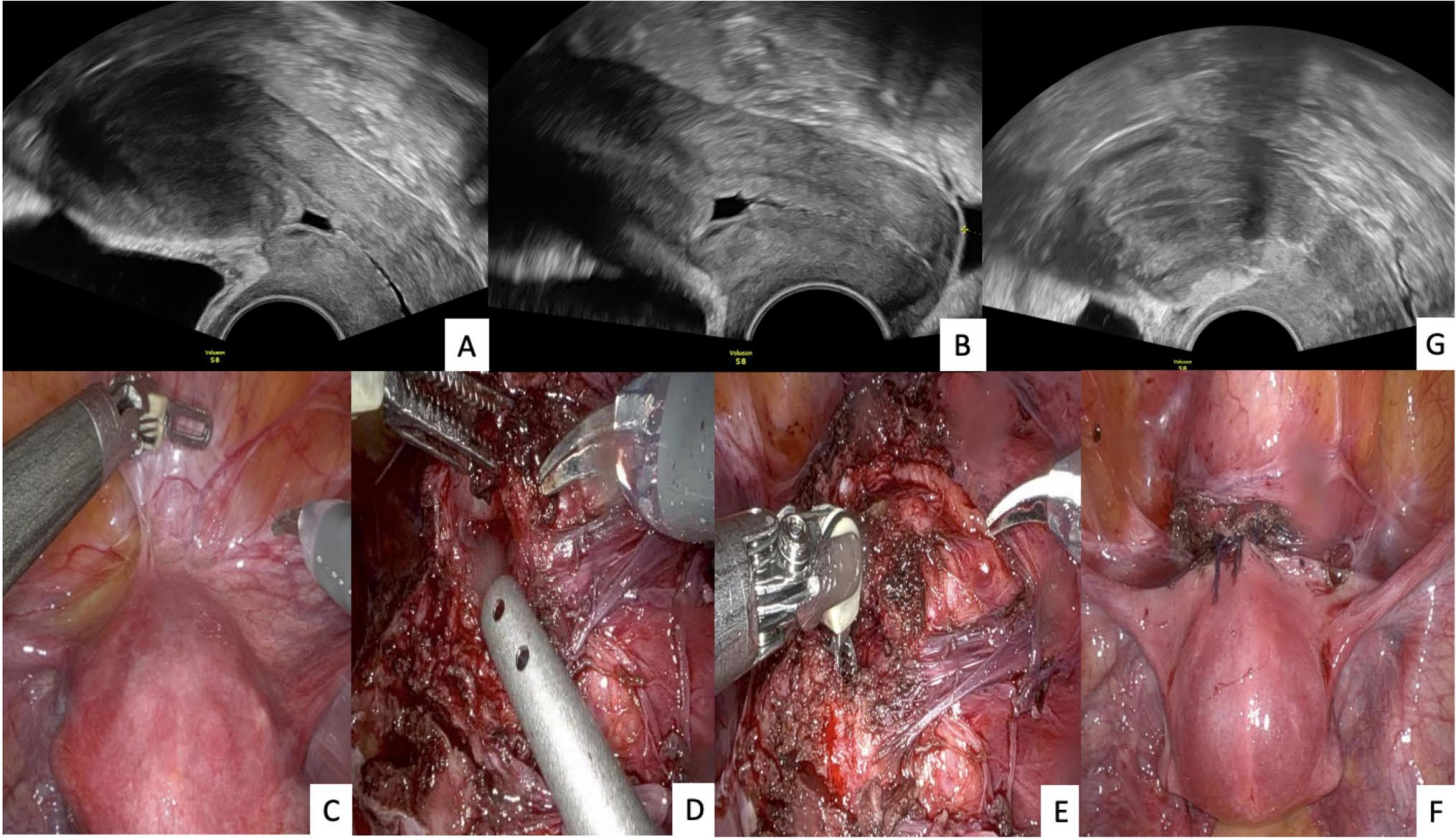
A+B Preoperative transvaginal ultrasound demonstrating an isthmocele. C: Intraoperative view prior to surgical dissection. D: Intraoperative view of the opened isthmocele following excision of the fibrotic niche tissue. E: Placement of a forced grasper within the cranial aspect of the uterine cavity to delineate the defect; the caudal wound margin dissected and mobilised from the urinary bladder (vesicouterine space). F: Completed surgical repair with monolayer closure of the myometrial defect. G: Postoperative transvaginal ultrasound demonstrating restoration of the anterior uterine wall with a residual myometrial thickness of 9 mm

Hysteroscopic visualisation of the isthmocele, followed by repositioning of the patient for the robot-assisted procedure. Careful dissection is performed to separate the bladder from the anterior uterine wall until the isthmocele and adjacent healthy cervical and myometrial tissue are fully exposed, thereby providing adequate anchoring points for subsequent suturing. The isthmocele is then excised until all wound margins demonstrate fresh bleeding, serving as confirmation that fibrotic scar tissue has been completely removed. Haemostasis is deliberately minimised to reduce the risk of thermal injury and tissue necrosis. Reconstruction is performed with interrupted Vicryl sutures, while the assistant elevates and repositions the uterus to facilitate tension-free approximation of the wound edges.

When concomitant macroscopic endometriosis was identified during surgery, all visible endometriotic lesions were excised during the same procedure.

### Statistical analysis

The analysis was conducted using SPSS 29.0.2.0 (IBM Corp., 2023, IBM SPSS Statistics for Windows, Version 29.0. Armonk, NY: IBM Corp.). Descriptive statistics were used to summarise patient characteristics, surgical data, and outcome variables. Categorical variables are presented as absolute numbers and percentages, while continuous variables are reported as medians with ranges or means with standard deviations (SD), as appropriate. Comparisons between the RAIR and LIR groups were performed using the Mann–Whitney *U* test for continuous variables and Fisher’s exact test for categorical variables, due to the small sample size. Within-group comparisons of preoperative and postoperative continuous variables were performed using the Wilcoxon signed-rank test. All tests were two-sided, and a *p*-value < 0.05 was considered statistically significant. Ethical approval was obtained from the local ethics committee at the University of Bonn (reference number 2026–173-BO). This study was registered in the German Clinical Trials Register (DRKS) under the number DRKS00038857 on April 15, 2026.

## Results

Between December 2019 and March 2026, 28 patients underwent CSD surgery at the Department of Gynaecology and Gynaecological Oncology at the University Hospital Bonn. Of these, 21 patients (75%) received RAIR, and 7 patients (25%) received LIR. Baseline characteristics are summarised in Table 1. There were no significant differences between RAIR and LIR except for the preoperative niche length, which was significantly larger in the laparoscopic group (*p* = 0.007), and the rate of anteflexed uterus, which was significantly higher in the laparoscopic group (*p* = 0.004).

**Table 1:** Patients’ characteristics and clinical presentation

	Total *n* = 28	RAIR *n* = 21	LIR *n* = 7	*p*-value
Age, years, median (range)	35 (21–44)	34 (21–42)	36 (21–44)	0.917
BMI, kg/m^2^, mean ± SD	27 ± 5	27 ± 6	28 ± 6	0.533
Number of caesarean sections, median (range)	1 (1–4)	1 (1–4)	1 (1–3)	0.836
Time since last caesarean section, years, median (range)	2 (0.5–16)	2 (0.5–15)	3 (1.5–16)	0.240
Flexion of the uterus, *n* (%)^a^				
Anteflexed	12 (42.9)	6 (28.6)	6 (85.7)	0.004*
Retroflexed	14 (50)	14 (66.7)	0	
N.A	2 (7.1)	1 (4.8)	1 (14.3)	
Preoperative maximum niche length, mm, median (range)	12.5 (2–33)	11 (2–32)	18.5 (12–33)	0.007
Preoperative maximum niche depth, mm, median (range)	5.5 (2–30)	5.5 (2–19)	6 (3–30)	0.501
Preoperative residual myometrial thickness, mm, median (range)	3.1 (0–8)	3 (0–8)	4 (1–5)	0.533
Abnormal uterine bleeding/spotting, *n* (%)^a^	13 (46.4)	11 (52.4)	2 (28.6)	1.00
Pelvic pain, *n* (%)^a^	11 (39.3)	10 (47.6)	1 (14.3)	0.586
Secondary infertility > 1 year, *n* (%)^a^	5 (17.9)	4 (19.0)	1 (14.3)	1.00
Caesarean scar pregnancy, *n* (%)^a^	3 (10.7)	2 (9.5)	1 (14.3)	0.371

^a^Percentages are calculated within each group

Mann–Whitney *U* test, except for: *Fisher’s exact test

Presenting symptoms and clinical indications were not mutually exclusive, as individual patients could report more than one symptom or indication. Moreover, symptoms were not a prerequisite for surgery; asymptomatic patients who elected surgical repair after comprehensive counselling are therefore not represented in the symptom categories. Consequently, the sum of the reported symptoms does not correspond to the total number of operated patients, and percentages do not add up to 100%

*RAIR* robotic-assisted isthmocele repair, *LIR* laparoscopic isthmocele repair, *SD* standard deviation, *CSD* caesarean scar defect, *N*.*A*. Not available/missing

The distribution of presenting symptoms was comparable between groups and is summarised in Table 1: eight patients in the RAIR group (38.1%) and three patients in the LIR group (42.9%) were asymptomatic, and the diagnosis of CSD was incidental. All five patients with secondary infertility were actively attempting conception before surgery.

Surgical data were comparable between the RAIR and LIR groups, with no significant differences in operating time or length of hospital stay (Table 2). No intraoperative or postoperative complications were observed in either group.

**Table 2:** Surgical data

	Total *n* = 28	RAIR *n* = 21	LIR *n* = 7	*p*-value
Operating time (skin-to-closure), min, median (range)	107 (62–229)	111 (62–172)	91 (64–229)	0.917
Intraoperative complications, *n* (%)	0	0	0	N.A
Postoperative complications, *n* (%)	0	0	0	N.A
Length of inpatient hospital stay, days, median (range)	3 (2–5)	2 (2–5)	3 (2–4)	0.376

Mann–Whitney *U* test, except *for Fisher’s exact test

*RAIR* robotic-assisted isthmocele repair, *LIR* laparoscopic isthmocele repair, *N*.*A*. Not available/missing

Histopathological assessment was performed in 23 patients (82%). Fibrotic and/or sclerotic scar tissue was the predominant finding of CSD, reported in 13 specimens (46.4%). Histological features compatible with endometriosis and chronic inflammatory changes were each identified in 9 cases (32.1%). No atypia or malignant changes were observed.

During the early study period, one LIR procedure was performed in each of 2019, 2020, and 2021. In 2022, one RAIR and two LIR procedures were performed, followed by three RAIR and one LIR procedure in 2023. In 2024, four RAIR and one LIR procedure were performed, whereas all procedures in 2025 and 2026 were robotic-assisted, with ten procedures in 2025 and three through March 2026. Overall, 18 of 28 procedures (64.3%) were performed from 2024 onwards. FU data were available for 23 patients (82%). The median FU duration was 4 months (Range 1–48).

One patient received recurrent LIR for persistent CSD after initial LIR. No procedure-related readmissions occurred during the FU period.

## Primary outcome

### Live-birth rate

Of the 23 patients included in the FU analysis, 8 patients attempted conception postoperatively (4 patients after RAIR and 4 patients after LIR). Among those trying to conceive, the live-birth rate was 75% (6 patients, 3 after RAIR and 3 after LIR, all at ≥ 36 + 0 weeks of gestation). The mode of delivery was repeat caesarean section in all cases, with a median interval of 7 months (Range 3–13 months) between MIIR and conception. Of the five patients with infertility ≥ 1 year preoperatively, one patient conceived and had a live birth, one patient tried to conceive without a live birth, and three patients did not try to conceive actively during the FU period.

## Secondary outcomes

### Obstetric complications

During repeat caesarean section, one case of uterine scar dehiscence and one case of placenta accreta spectrum disorder were observed, both after RAIR (each 3.6%). No cases of uterine rupture were observed. The mode of delivery was elective repeat caesarean section in all cases.

### Subjective patient-reported outcomes

Among 13 patients reporting abnormal uterine bleeding or postmenstrual spotting preoperatively, 6 women described marked improvement or complete resolution of symptoms at last FU (5 patients after RAIR, 1 patient after LIR). None of the patients described worsening of abnormal uterine bleeding or spotting. Pelvic pain was present preoperatively in 11 patients. Postoperatively, 3 of these patients reported complete resolution of pain; all patients received RAIR. No patient reported deterioration of pain.

### Objective imaging outcomes

Postoperative transvaginal ultrasound data were available for 23 patients. There were no differences between the RAIR and LIR groups in the decrease in niche length and depth or in the increase in myometrial thickness (Table 3). In the RAIR group, a significant decrease in niche length and niche depth as well as a significant increase in myometrial thickness were observed between pre- and postoperative. In the LIR group, the trends did not reach significance (Table 4).

**Table 3:** Postoperative outcome compared between RAIR and LIR

	Total *n* = 23	RAIR *n* = 19	LIR *n* = 4	*p*-value
Decrease of niche length, mm, median (range)	9 (+ 5–33)	8 (+ 5–32)	18.5 (1–33)	0.162
Decrease of niche depth, mm, median (range)	6 (+ 5–30)	6 (+ 2–15)	8 (+ 5–30)	0.654
Increase myometrial thickness, mm, median (range)	2.7 (− 2.6–14)	3.5 (− 2.6–14)	0.9 (− 1–10)	0.667
Postoperative niche length, mm, median (range)	3 (0–19)	3 (0–11)	0 (0–19)	0.505
Postoperative niche depth, mm, median (range)	1 (0–12)	2 (0–4)	0 (0–12)	0.543
Postoperative residual myometrial thickness, mm, median (range)	5.7 (2–17)	6 (2–17)	4.9 (2–11)	0.611

Mann–Whitney *U* test for comparing RAIR vs. LIR

*RAIR* robotic-assisted isthmocele repair, *LIR* laparoscopic isthmocele repair, *CSD* caesarean scar defect, *N*.*A*. Not available/missing

**Table 4:** Outcome compared within groups between pre- and postoperative

	RAIR preoperative	RAIR postoperative	*p*-value	LIR preoperative	LIR postoperative	*p*-value
Maximum niche length, mm, median (range)	11 (2–32)	3 (0–11)	< 0.001	18.5 (12–33)	0 (0–19)	0.068
Maximum niche depth, mm, median (range)	5.5 (2–19)	2 (0–4)	< 0.001	6 (3–30)	0 (0–12)	0.273
Residual myometrial thickness, mm, median (range)	3 (0–8)	6 (2–17)	0.006	4 (1–5)	4.9 (2–11)	0.285

Wilcoxon test for comparing RAIR pre- and postoperative and LIR pre- and postoperative

*RAIR* robotic-assisted isthmocele repair, *LIR* laparoscopic isthmocele repair, *CSD* caesarean scar defect

#### Concomitant endometriosis

Concomitant macroscopic endometriosis was identified in three patients, all of whom underwent RAIR. All visible endometriotic lesions were excised during RAIR. One of these patients was lost to FU, while the remaining two did not actively attempt conception postoperatively. Consequently, none of the patients with surgically treated endometriosis during RAIR contributed to the reproductive outcome analysis of women attempting conception.

## Discussion

CSD are increasingly recognised as a clinically relevant condition associated with abnormal uterine bleeding, pelvic pain, and impaired fertility [1]. Despite growing awareness, evidence guiding optimal surgical management—particularly regarding fertility-preserving approaches—remains limited, especially after a robot-assisted approach. We evaluated reproductive, clinical, and imaging outcomes after MIIR and provided additional data supporting its safety and potential effectiveness in a selected patient population and according to the specific minimally invasive approach.

The primary objective of this study was to assess the live-birth rate after MIIR. Among women attempting conception during FU, 75% achieved a live birth. Data from previously published observational studies using a robot-assisted approach are scarce; the largest study, with 33 patients, reports a live-birth rate of 70% [17].

Although the absolute number of pregnancies in our cohort is small, these findings are clinically meaningful given the high prevalence of reduced residual myometrial thickness and retroflexed uteri in our population—both factors previously associated with impaired fertility and adverse obstetric outcomes [18, 19 ]. In Germany, the reimbursement for isthmocele repair remains consistent regardless of the surgical technique employed. However, the costs associated with robot-assisted procedures are significantly higher. Should it be determined that this approach, with its relatively lower learning curve, yields the most favourable outcomes, it would be advisable to consider an appropriate reimbursement structure for the use of robot-assisted techniques, in line with the principles of FEMTECH, to encourage the broader adoption of this advanced method.

Our secondary outcomes further support the clinical value of MIIR. Symptom relief was observed in the majority of symptomatic patients, particularly with respect to abnormal uterine bleeding and pelvic pain. Bleeding symptoms improved in half of the patients, and pelvic pain improved or resolved in all patients. These findings align with prior reports demonstrating that surgical correction of the isthmocele can reduce symptoms such as menstrual blood retention and spotting [11]. The absence of symptom deterioration in most patients underscores the favourable risk–benefit profile of the procedure. Objective imaging outcomes showed a substantial increase in residual myometrial thickness and a reduction or resolution of niche dimensions in the majority of patients. Median residual myometrial thickness increased from 3 mm preoperatively to 6 mm postoperatively, consistent with previous reports suggesting that surgical reconstruction can restore myometrial continuity [12, 17 ]. Although a universally accepted threshold for “safe” residual myometrial thickness has not been established, several studies suggest that thicknesses below 3 mm are associated with higher risks of symptoms and adverse events, suggesting an indication for surgical repair [11, 12, 14 ]. Enhanced instrument articulation in especially robotic surgery facilitates tissue approximation and full-thickness suturing, with shorter learning curves, as shown by significant postoperative improvements in residual myometrial thickness, symptom relief, and favourable reproductive outcomes following robotic repair [12, 13 ].

There are several limitations. The retrospective design and small sample size of our study limit the generalisability of our findings and preclude robust inferential conclusions. Due to a median FU duration of only 4 months, pregnancy rates and long-term obstetric outcomes may be underestimated. The absence of predefined FU intervals, the inability to obtain an updated examination in all patients, and the reliance on the most recent available clinical records resulted in heterogeneous FU durations and may have led to incomplete ascertainment of long-term reproductive and obstetric outcomes. In addition, not all women with a desire to conceive actively attempted conception during FU, reflecting real-world circumstances and complicating the interpretation of fertility outcomes of this study, since patients are counselled to prevent conception for 3 to 6 months after MIIR. Since, e.g. a double-layer uterine closure, dilation of the inner cervix, and interrupted sutures instead of continuous suturing during the initial caesarean section are discussed as risk-reducing techniques for CSD, standardisation of uterine closure techniques and comparable randomised studies are needed to further evaluate this hypothesis [20–22]. Moreover, the small number of patients with concomitant endometriosis, none of whom contributed to the pregnancy-attempt cohort, precluded a meaningful analysis of reproductive outcomes after MIIR according to endometriosis status. Another limitation is that although niche length, depth, and residual myometrial thickness were assessed in the sagittal plane, niche width in the transverse plane and adjacent myometrial thickness were not routinely documented. Therefore, the ultrasound assessment did not include all measurements recommended by the Delphi consensus, which may limit comprehensive morphological comparison with other studies [16].

Despite these limitations, our study adds to the growing body of evidence supporting MIIR and especially RAIR as a safe and effective option for selected patients with CSD, particularly those desiring future fertility and/or experiencing symptoms such as abnormal uterine bleeding. The favourable reproductive outcomes, high patient satisfaction, symptom improvement, and reassuring obstetric safety profile observed in this cohort underscore the potential role of especially RAIR in comprehensive CSD management. Larger prospective studies with longer FU and standardised outcome reporting are warranted to further define patient selection criteria and to compare robotic repair with other minimally invasive techniques.

## Conclusions

RAIR appears to be a safe and effective minimally invasive option for selected women with CSD, offering favourable reproductive outcomes, symptom improvement, and restoration of myometrial integrity. Further prospective, adequately powered studies with longer FU are required to define optimal patient selection, standardise surgical techniques, and confirm long-term obstetric safety.

## Data Availability

The dataset and materials used in this study are available from the corresponding author upon reasonable request.

## Abbreviations

BMI: Body mass index
CSD: Caesarean scar defect
FU: Follow-up
LIR: Laparoscopic isthmocele repair
MIIR: Minimally invasive isthmocele repair
N.A.: Not available
RAIR: Robotic-assisted isthmocele repair
SD: Standard deviation
